# Evaluation of the functional outcome and mobility of patients after stroke depending on their cognitive state

**DOI:** 10.1038/s41598-024-52236-8

**Published:** 2024-01-17

**Authors:** Zbigniew Guzek, Wioletta Dziubek, Małgorzata Stefańska, Joanna Kowalska

**Affiliations:** 1 Department of Neurological Rehabilitation, University Hospital in Zielona Góra, 65-046 Zielona Gora, Poland; 2 Faculty of Physiotherapy, Wroclaw University of Health and Sport Sciences, Paderewskiego 35 Street, 51-612 Wrocław, Poland

**Keywords:** Diseases, Medical research, Neurology

## Abstract

The study aimed to analyze the functional outcome and mobility in stroke patients depending on their cognitive state. 180 patients after first stroke were divided into four groups: 48 patients without symptoms of cognitive impairment (G1); 38 with mild cognitive impairment without dementia (G2); 47 with mild dementia (G3); 47 with moderate dementia (G4). The Mini Mental State Examination (MMSE), Barthel Index (BI), Sitting Assessment Scale (SAS), Berg Balance Scale, Trunk Control Test and Test Up & Go were used. The tests were carried out at the time of admission to the ward (T1) and at the time of discharge (T2). A statistically significant improvement was demonstrated in all parameters in almost all groups. No significant difference was observed only in groups G1 and G4 in SAS head. Statistically significant differences in BI results in T2 between groups G1 and G4 were noted. The lowest change in BI was observed in the G4. Regression analysis showed that MMSE and BI at T1 and MMSE score at T2 explained the functional status at T2. Cognitive dysfunction at the time of admission to the ward and discharge may determining the patient's functional status at the time of discharge from the ward.

## Introduction

Cognitive dysfunction is observed in approximately 66% of stroke patients within 6 months of stroke and 70% in the first year after stroke^1–3^.

Many authors indicate that stroke patients are at increased risk of developing cognitive impairment (CI), and the occurrence of CI may progress to developing dementia^1,3–5^. One-third of patients after stroke have a significant degree of CI within the first month after stroke^6^. Research results by Liao et al. indicate that the occurrence of CI among patients after ischemic stroke was 52.4%, 35.5%, and 34.2% at 2 weeks, 3 months and 12 months^2^. Sexton et al. state in their analysis that 4 in 10 patients after stroke display a CI but not dementia^7^.

Research confirms that the presence of cognitive disorders is an adverse prognostic factor in patients after a stroke: it increases the risk of disability, significantly complicates the rehabilitation process, affects the effectiveness of the rehabilitation and recovery process, and worsens their quality of life^6^. Additionally, the combination of stroke and CI increases the severity of problems with basic and instrumental activities of daily living^1^.

Such negative effects of stroke mean that at the beginning of a rehabilitation process, one should focus not only on the patient's functional status and physical deficits but also on their cognitive state. The cognitive state of patients at the time of admission to the ward may be one of the many factors influencing the rehabilitation outcome^8,9^. Kowalska et al. show that the group of elderly patients with co-existing dementia had lower physiotherapy efficacy than that in patients without dementia. Moreover, the authors emphasize that the level of cognitive status at the time of admission to the rehabilitation ward (not functional status) significantly impacted the rehabilitation outcome^10,11^.

The studies conducted so far mainly concern patients with mild cognitive impairment (MCI) or mild dementia. In many research projects (including stroke patients), the presence of CI or dementia is the criteria for excluding patients from participation in research^3,8,12–14^. Therefore, there are few reports on post-stroke patients with moderate-to-severe dementia. Few of them confirm that stroke patients with severe cognitive deficits have the highest risk of rehabilitation failure^10^.

Therefore, the study aimed to assess the functional outcome and mobility in patients after stroke depending on their cognitive state at the time of admission to the rehabilitation ward. It was hypothesized that the functional outcome in the group of patients after a stroke with a suspicion of moderately advanced dementia would be the lowest, and the existing cognitive dysfunctions and dementia are the factors determining the patient's functional status at the time of discharge from the ward.

This observational study can contribute to a deeper reflection on the scale of the problem of occurrence the cognitive impairment and dementia in patients after a stroke (including those patients with moderate dementia) staying at rehabilitation ward. The results may have significance in planning and changing the patterns of procedure in centers, which providing rehabilitation process for people after stroke.

This is an important topic due to the high incidence of cognitive disorders in stroke patients and the need to support medical practitioners in effective, evidence-based work with stroke patients.

## Materials and methods

### Participants

This study was carried out at the Neurological Rehabilitation Unit of the Department of Rehabilitation of the University Hospital in Zielona Góra, Poland, with the consent of the head of the department and under the ethical and legal supervision of the Bioethics Committee of the Wroclaw University of Health and Sport Sciences, Poland (reference No. 16/2021). The study was conducted following the Helsinki Declaration. All patients were informed about the aim and methods of the study and the procedures used.

The study group consisted of patients after stroke who were admitted to the rehabilitation ward and who were satisfied with the following criteria for inclusion: a written informed consent to participate in the study, first stroke incident, ischemic or hemorrhagic type of stroke, patients with hemiplegia, lack of pre-stroke dementia (according medical records), patients admitted from the hospital neurological unit and the possibility of performing the Mini-Mental State Examination (MMSE). The exclusion criteria were also established: aphasia, patients with diplegia or monoplegia, refusal to participate at any stage of the study, taking medications that may affect cognitive functioning, and the presence of severe mental disorders in the medical records (e.g., consciousness disorders, depression, psychosis, schizophrenia disease).

Finally, the study group covered 180 patients with a mean age of 69.03 (± 12.3), 75 women and 105 men, mostly (89%) after ischemic stroke and 70% concerning the brain's right hemisphere.

The patients were divided into four groups according to their cognitive status (the MMSE results):

- Group 1 (G1)—48 patients without cognitive impairment and dementia (MMSE = 30–27 points);
- Group 2 (G2)—38 patients with mild cognitive impairment (MCI) and without dementia (MMSE = 26–24);
- Group 3 (G3)—47 patients with suspected mild dementia (MMSE = 23–19);
- Group 4 (G4)—47 patients with suspected moderate dementia (MMSE = 18–11).

The characteristics of the patients are presented in Tables 1 and 2.

**Table 1:** Characteristics of patients in all study groups and subgroups (χ^2^ test).

Baseline characteristics	All N = 180		G1 N = 48		G2 N = 38		G3 N = 47		G4 N = 47		χ^2^ test
	N	%	N	%	N	%	N	%	N	%	*p*
Gender											
Female	75	41.7	16	33.3	17	44.7	17	36.2	25	53.2	0.1975
Male	105	58.3	32	66.7	21	55.3	30	63.8	22	46.8	
Education											
Secondary and higher	107	59.4	25	52.1	19	50.0	29	61.7	34	72.3	0.1204
Primary and vocational	73	40.6	23	47.9	19	50.0	18	38.3	13	27.7	
Marital status											
Single (widow(er), unmarried)	96	53.3	26	54.2	23	60.5	24	51.1	23	48.9	0.7373
Married	84	46.7	22	45.8	15	39.5	23	48.9	24	51.1	
Type of stroke											
Ischemic	160	88.9	41	85.4	35	92.1	43	91.5	41	87.2	0.6971
Haemorrhagic	20	11.1	7	14.6	3	7.9	4	8.5	6	12.8	
Lesion location											
Right hemisphere	126	70.0	44	91.7	32	84.2	33	70.2	17	36.2	< 0.0001*
Lefthemisphere	54	30.0	4	8.3	6	15.8	14	29.8	30	63.8	

*p < 0.05.

**Table 2:** Characteristics of patients in all study groups and subgroups (ANOVA).

Characteristic	Group	Median	IQR	ANOVA *p*
Age	G1	71.00	14.5	0.7922
	G2	70.00	15.0	
	G3	69.00	14.0	
	G4	66.00	16.0	
MMSE (T1)	G1	28.00	2.0	< 0.0001*
	G2	25.00	2.0	
	G3	22.00	2.0	
	G4	15.00	4.0	
MMSE (T2)	G1	27.00	4.5	0.4237
	G2	27.00	4.0	
	G3	27.00	7.0	
	G4	26.00	6.0	
Time since stroke to admission to the ward [days]	G1	10.00	3.5	0.0002*
	G2	13.50	9.0	
	G3	14.00	8.0	
	G4	13.00	8.0	
Length of stay in the ward [days]	G1	55.50	57.5	0.0146*
	G2	55.50	68.0	
	G3	66.00	68.0	
	G4	39.00	35.0	

*p < 0.05.

### Measurement tools

The Mini Mental State Examination (MMSE), the Barthel Index (BI), the Sitting Assesment Scale (SAS), the Berg Balance Scale (BBS), the Trunk Control Test (TCT) and the Test Up & Go (TUG) were used. Additionally, sociodemographic and clinical data were collected based on existing medical records.

The polish version of the MMSE was developed by Stańczak and it assesses the patients’ cognitive function^15^.^.^ The patient can score a maximum of 30 points and the result below 24 points suggests dementia. The results were calculated using the formula published by Mungas et al., which considered the age and education level of participants^16^.

The BI assesses the patient’s functional status, especially the basic activities of daily living. The patients can score a maximum of 100 points. Results from 0–20 points indicate a severe condition, 21–85 a medium-heavy condition, and 86–100 points indicate a mild condition^17^.

The SAS assesses sitting ability by analysing head, trunk and foot control, arm and hand function. The patient's sitting balance is scored as: 4—able to perform the above tests without any physical assistance; 3—able to maintain a static position without difficulty, however requiring assistance, especially in righting from the hemiplegic side; 2—able to maintain a static position but requiring assistance in all righting tasks; and 1—unable to maintain a static position^18,19^.

The BBS evaluates a patient’s static and dynamic balance and consists of 14 tasks which include: sitting unsupported, standing unsupported, standing with eyes closed, standing with feet together, standing on one foot, turning to look behind, retrieving an object from the floor, tandem standing, reaching forward with an outstretched arm, sitting to standing, standing to sitting, transferring, and turning 360° and then stepping on a stool. A score from 0 to 4 is given for each task. The patients can score a maximum of 56 points. The higher the total score, the better the balance^20^.

The TCT assesses trunk movement of patient: rolling on a patient’s strong and weak sides, sitting up from lying down, and sitting in a balanced position on the edge of the bed, with feet off the ground. Patient can get the following scores: 0—unable to perform movement without assistance; 12—able to perform movement, but in an abnormal way; and 25—able to complete movement normally. The patients can score a maximum of 100 points^21^.

The TUG test assesses the patient’s functional ability and fall risk. The patient has to stand up from a chair, walk 3 m, turn around, walk back to the chair, and sit down. The timing of the test begins at the word “go,” and ends when the patient is seated. The patient can use supporting equipment during the test^22,23^.

The above tests were conducted at two testing points: T1(the initial assessment), on the first day of a patient’s admission to the ward; T2 (the final assessment), on the last day of their stay on the ward. The total measurement time lasted approximately 70–90 min (with short breaks between subsequent tests).

The assessment of the functional status of all patients, upon admission to the ward and discharge, was performed by the one physiotherapist, who was not involved in the rehabilitation process. Patients after initial assessment were met with a physiotherapist with whom they started work. He informed them about existing rules and schedule of their day including rehabilitation programme (main goals, time and frequency etc.).

All patients took part in a regular rehabilitation programme which lasted from Monday to Friday for about 150 min per day and 90 min on a Saturday. This programme was carried out by a doctor’s instructions and was dependent on the patient’s functional status. It included individual exercises with a physiotherapist (120 min, including elements of Proprioceptive Neuromuscular Facilitation method (PNF) and Bobath method), and learning and improving gait (30 min, e.g. walking on a flat and uneven surface, walking on a special learning track and learning to walk up and down stairs). Before admission to the rehabilitation ward, all patients had early post-stroke rehabilitation in the hospital ward.

### Data analysis

Using the Shapiro–Wilk test, the normal distribution of most variables measured in the quantitative scale was not confirmed. Descriptive statistics were calculated. The median was used to measure the central tendency of ordinal and quantitative variables, and the quartile range (IQR) was used to measure dispersion. The significance of differences between the groups was tested by Kruskal- Wallis ANOVA test and the χ^2^ test. The Wilcoxon test was used to check the significance of differences between the results in the initial (T1) and final assessment (T2).

Kruskal–Wallis ANOVA was also used to assess the significance of differences between the results obtained in the four study groups, considering the measurement number. When the analysis of variance showed statistical significance, the test of multiple comparisons of mean ranks was used as a post hoc test Dunn Bonferroni-Holm.

Additionally, multivariate regression analysis was performed to identify associations between the BI scores at the patients' discharge (T2) results and the selected parameters. To determine the quantity of the effect of differences between the examined groups, the corrected Cohen's d-test was used. The interaction effect size was calculated by Eta squared (η2) and then transformed to Cohen's d value. Values of Cohen's d-test ≥ 0.8 proved the high strength of the observed effect^24^. Effect size for the Wilcoxon test was checked using the biserial correlation coefficient for matched pairs (rc) ranging from -1 to 1. The significance level was assumed at *p* < 0.05. All the calculations were made in Statistica 13.1.

## Results

The 4 groups of patients did not differ in terms of gender, education, marital status, type of stroke or age. A statistically significant difference was noted for lesion location, time since the stroke and the length of stay in the ward (Tables 1, 2). The analyzed groups differed significantly in the results of the MMSE in the initial test (T1) because the division into groups was made based on this parameter. However, at T2, there was no statistically significant difference between the study groups (Table 2).

Comparing the initial (T1) and final (T2) examinations, a statistically significant improvement was demonstrated in all the examined parameters (SAS, BI, BBS, TCT) in almost all groups. No significant difference was observed only in groups G1 and G4 in SAS head (Table 3).

**Table 3:** Studied parameters at T1 and T2 (Wilcoxon test results).

Group	Tests	T1		T2		Wilcoxon	rc
		Median	IQR	Median	IQR	p	
G1 N = 48	SAS head	4.00	0.00	4.00	0.00	0.0519	0.73
	SAS trunk	3.00	1.00	4.00	0.00	< 0.0001*	0.87
	SAS arm control	3.00	2.00	4.00	1.00	< 0.0001*	0.87
	SAS hand function	3.00	2.50	4.00	1.00	< 0.0001*	0.87
	BI	10.00	25.00	100.00	20.00	< 0.0001*	0.87
	BBS	20.00	29.00	50.50	12.00	< 0.0001*	0.87
	TCT	100.00	26.00	100.00	0.00	< 0.0001*	0.87
G2 N = 38	SAS head	4.00	0.00	4.00	0.00	0.0064*	0.76
	SAS trunk	3.00	1.00	4.00	0.00	< 0.0001*	0.88
	SAS arm control	3.00	3.00	4.00	1.00	< 0.0001*	0.87
	SAS hand function	2.00	2.00	4.00	1.00	< 0.0001*	0.87
	BI	27.50	45.00	87.50	25.00	< 0.0001*	0.87
	BBS	20.50	25.00	46.00	16.00	< 0.0001*	0.87
	TCT	100.00	39.00	100.00	0.00	0.0002*	0.88
G3 N = 47	SAS head	4.00	0.00	4.00	0.00	0.0277*	0.90
	SAS trunk	4.00	1.00	4.00	0.00	0.0049*	0.68
	SAS arm control	3.00	3.00	4.00	1.00	0.0003*	0.88
	SAS hand function	2.00	2.00	4.00	1.00	0.0001*	0.88
	BI	35.00	50.00	95.00	70.00	< 0.0001*	0.84
	BBS	22.00	29.00	47.00	23.00	< 0.0001*	0.78
	TCT	87.00	51.00	100.00	0.00	0.0038*	0.75
G4 N = 47	SAS head	4.00	0.00	4.00	0.00	0.1088	0.93
	SAS trunk	4.00	1.00	4.00	0.00	0.0180*	0.89
	SAS arm control	3.00	2.00	4.00	1.00	0.0077*	0.89
	SAS hand function	3.00	2.00	4.00	1.00	0.0077*	0.89
	BI	50.00	60.00	80.00	30.00	0.0003*	0.88
	BBS	27.00	29.00	48.00	20.00	0.0016*	0.76
	TCT	100.00	26.00	100.00	0.00	0.0180*	0.89

SAS, Sitting Assesment Scale; BI, Barthel Index; BBS, Berg Balance Scale; TCT, Trunk Control Test; T1, initial assessment; T2, final assessment; IQR, Interquartile Range; SD, Standard Deviation; G1, patients without dementia; G2, patients with MCI; G3, patients with mild dementia; G4, patients with moderate dementia; *p < 0.05.

Comparative analysis of the examined parameters at the time of admission to the ward (T1) showed statistically significant differences only in the BI results between the G1 and other groups. On the other hand, at T2, statistically significant differences in BI results were noted between groups G1 and G4. Patients with moderate dementia had significantly worse functional status than patients with intellectual ability (Table 4).

**Table 4:** Comparison of the initial and final examinations between the four groups (ANOVA—test results Post-hoc factor analysis).

Tests	ANOVA *p*	T 1						d’ Cohen
		G1 versus G2	G1 versus G3	G1 versus G4	G2 versus G3	G2 versus G4	G3 versus G4	
SAS head	0.4304	NS	NS	NS	NS	NS	NS	0.08
SAS trunk	0.6606	NS	NS	NS	NS	NS	NS	0.18
SAS arm	0.4288	NS	NS	NS	NS	NS	NS	0.07
SAS hand	0.2786	NS	NS	NS	NS	NS	NS	0.14
BI	0.0001*	0.0322*	0.0036*	< 0.0001*	NS	NS	NS	0.78
BBS	0.6463	NS	NS	NS	NS	NS	NS	0.18
TCT	0.1509	NS	NS	NS	NS	NS	NS	0.23

	ANOVA *p*	T 2						d’ Cohen
		G1 versus G2	G1 versus G3	G1 versus G4	G2 versus G3	G2 versus G4	G3 versus G4	
SAS head	0.7124	NS	NS	NS	NS	NS	NS	0.19
SAS trunk	0.6224	NS	NS	NS	NS	NS	NS	0.17
SAS arm	0.8181	NS	NS	NS	NS	NS	NS	0.22
SAS hand	0.2786	NS	NS	NS	NS	NS	NS	0.07
BI	0.0107*	NS	NS	0.0241*	NS	NS	NS	0.39
BBS	0.5322	NS	NS	NS	NS	NS	NS	0.14
TCT	0.1121	NS	NS	NS	NS	NS	NS	0.26

SAS, Sitting Assessment Scale; BI, Barthel Index; BBS, Berg Balance Scale; TCT, Trunk Control Test; T1, initial assessment; T2, final assessment; G1, patients without dementia; G2, patients with MCI; G3, patients with mild dementia; G4, patients with moderate dementia; *p < 0.05; NS, not statistically significant.

Additionally, the change in BI over time was calculated. This was the difference in the Barthel Index between T2 and T1 (BI in T2—BI in T1). A significant increase (improvement) in the results was observed in each of the study groups. The analysis showed that the lowest change was observed in the G4 group (with suspected moderate dementia). The observed differences between the groups were statistically significant, except for the comparison of the G3 and G4 groups (Table 5).

**Table 5:** The change in BI over time (difference in the Barthel Index between T2 and T1).

	Median	IQR	ANOVA		post-hoc test			Cohen's d
			p		G1	G2	G3	
G1	70.00	25.00	< 0.0001*		p	p	p	
G2	52.50	35.00		G2	0.0346*			1.18
G3	35.00	30.00		G3	< 0.0001*	0.0032*		
G4	30.00	20.00		G4	< 0.0001*	0.0096*	0.6675	

G1. patients without dementia; G2. patients with MCI; G3. patients with mild dementia; G4. patients with moderate dementia; *p < 0.05;

In the case of the TUG test, qualitative data analysis was performed. Due to the small number of subgroups, the analysis was performed by dividing patients into only 2 subgroups: without dementia (MMSE ≥ 24) and with suspected dementia (MMSE < 24). The analysis showed a statistically significant improvement in both non-demented and demented patients.

It seems significant that 3/4 of the patients without dementia and 40% of the patients with dementia who were unable to perform the test in T1 but performed it on their own without the aid of gait aids in T2. At the end of therapy, only 7 of 86 non-demented patients and 19 of 94 demented patients could not perform the TUG test. At the same time, 58 and 62 were unable to perform the test during the initial examination, respectively (Table 6).

**Table 6:** Qualitative comparison of the performance of the TUG test at T1 and T2 in the group of patients without dementia (MMSE ≥ 24) and with dementia (MMSE < 24).

TUG test	Patients without dementia N = 86				Patients with dementia N = 94			
	T1		T2		T1		T2	
	N	%	N	%	N	%	N	%
Independent walking	11	12.79	46	53.49	20	21.28	53	56.38
Done with walking frame	13	15.12	21	24.42	11	11.70	11	11.70
Done with stick	4	4.65	12	13.95	1	1.06	11	11.70
Not done	58	67.44	7	8.14	62	65.96	19	20.21
χ^2^ test p	0.0099*				0.0041*			
d Cohen	1.1619				1.2416			

TUG, test Up & Go; T1, initial assessment; T2, final assessment; *p < 0.05.

Multivariate regression analysis showed that MMSE and BI scores at admission to the ward (T1) and MMSE at T2 have a significant effect on the BI scores at patients' discharge from the ward (T2) (Table 7).

**Table 7:** Multivariate regression analysis exploring the effects of the studied parameters on BI scores at T2.

Parameters	Co.B	± 95% CI	*p*
Age	-0.21	-0.48–0.07	0.1505
Type of stroke	4.12	-6.23–14.61	0.4286
Lesion location	-0.21	-8.18–7.77	0.9602
MMSE (T1)	1.01	0.27–1.73	0.0073*
MMSE (T2)	1.98	1.23–2.73	< 0.0001*
BI (T1)	2.47	1.70–3.24	< 0.0001*
R^2^_Adj_	0.43		
p	< 0.0001*		

MMSE, Mini Mental State Examination; BI, Barthel Index; T1, initial assessment; T2, final assessment; Co. B, slope coefficient; CI, confidence interwal;*p < 0.05.

## Discussion

Effective rehabilitation, i.e. improvement of the patient's functional status is an integral part of the recovery process and regaining independence of patients after a stroke. Unfortunately, many factors hinder this process^8,10,12,13^. One of them may be cognitive disorders^25–28^.

In this study the comparison of the studied groups of patients with different cognitive functions at the time of admission to the ward (T1) showed no significant differences in demographic variables. Still, there were significant differences in the time from stroke and the length of stay in the ward. At the time of admission, patients without CI were characterized by a significantly shorter time elapsed since the stroke and a substantially longer length of stay in the rehabilitation ward compared to patients with dementia. Similar results were reported by Kowalska et al.^29^. Also, Tornes et al. showed that dementia influences the patient's acute hospital length of stay^30^. According to Liu et al., dementia was the most notable length of stay-specific and cost-specific comorbidities among patients after stroke^31^.

The assessment of the functional status of the examined patients showed a significant differences in the functional status of patients with moderate dementia compared to patients without cognitive impairment. At the time of admission to the ward patients without cognitive impairment had worse functional status. However, at the time of discharge from the ward, patients with moderate dementia were characterized by a significantly worse functional status compared to patients with intellectual ability. Also, in a group of patients with suspected moderate dementia the change in functional status over time (difference between BI inT2 and BI in T1) was the lowest. Similar results were published by Sawyer et al. Patients with CI were more likely to experience withdrawal of care during hospitalization, and for survivors, had greater disability and lower BI scores, especially after hemorrhagic stroke^32^. In the studies of Oros et al. analysed a correlation between the MMSE and activities of daily living of patients after a stroke. They noted that patients with CI were more dependent^33^.

Other researchers emphasize that the occurring disorders of cognitive functions in patients after a stroke (post-stroke dementia) are the cause of addiction and disability^34^. It is also an important reason for the poor prognosis in patients after stroke with motor and speech dysfunction^27^. According to Lee et al., CI after stroke can increase the limitations of activity of daily living. Patients after a stroke with CI had the highest prevalence of disabilities in basic and instrumental activities of daily living^1^. However, the author points out that the inability to perform certain activities may result from paresis, not CI.

It is worth mentioning that patients with suspected moderate dementia stayed in the ward for the shortest time. Unfortunately, this is a common situation noted, for example, by Mizrahi et al.^25^. Perhaps communication problems, lack of an active attitude in the rehabilitation process, constant need for patient motivation, memory and attention problems make working with them difficult and require greater commitment from the medical staff. These patients are often transferred to long-term care centers after early rehabilitation. This is confirmed by the studies of Sibolt et al., in which the authors emphasize that post-stroke dementia is associated with shorter survival time and earlier permanent institutionalization compared to patients without post-stroke dementia^35^.

Unfortunately patients with dementia derive less benefit from standard rehabilitation, and failure to take into account the cognitive state by medical staff additionally hinders the rehabilitation process. It affects the final results of the patient's stay in the ward^10^.

Another explanation why they are not able to perform their activities of daily living are episodic or working memory, executive, and instrumental function disturbances^36^. According Yaghi et al. also cognitive deficits such as visuospatial or executive dysfunctions may limit functional independence^37^.

Nevertheless, obtained results noted that patients with CI and dementia could be successful. Comparison of the initial and final tests showed statistically significant improvement in all tested parameters (SAS, TCT, BBS, BI), also in the group of patients with moderate dementia. Additionally,the analysis of the TUG test showed that 19 out of 94 patients with dementia were unable to perform the TUG test in this group at the time of discharge. In contrast, in the initial test, the inability to perform the test was observed in as many as 62 people. Many authors have noted that exercise applied in stroke patients can improve their cardiovascular fitness, walking ability, and muscle strength^38–41^. Furthermore, research suggests that exercise may improve cognitive status as some executive functioning, memory and other health-related quality of life for post-stroke patients^5,42^. This helps stroke patients achieve improving the functional status and independence^10^. However, in case of patients with dementia, this requires a longer time and, unfortunately, does not guarantee a return to full functional efficiency. And even then, the occurring symptoms of dementia at the intermediate level are a premise for implementing more care on the part of caregivers or providing institutional care^43^.

The results indicate a significant relationship between the cognitive status and the functional status of patients after stroke. The regression results also confirm this. In the present study, the MMSE and BI scores at admission to the ward and also the MMSE score at T2 explained as much as 43% of the functional status at discharge. This confirms that cognitive functions and functional status of stroke patients (at admission to the ward) are predictors of functional status at discharge. Similar conclusions were presented by Perez et al. They confirmed that worse cognitive ststus at admission was significantly associated with a lower probability of returning home with functional improvement^44^. Also, Sharma et al., emphasized that cognitive state is a significant, independent predictor for functional status during the early phase of post-stroke recovery and at the follow-up^45^.

It is also worth emphasizing the dynamics of changes in cognitive functions in patients after a stroke. The obtained results indicate an improvement in the cognitive state of the examined patients at the time of discharge from the ward. This improving can be spontaneous, due to recanalisation or cerebral plasticity as a result of adjacent or contralesional brain regions taking over cognitive tasks^46^. On the other hand regular exercise can increase cerebral blood flow, improve oxygen consumption, and promote brain cell regeneration in the encephalic regions related to cognitive function^47^.

Nevertheless, Mijajlowić et al. point out that cognitive impairment is gradually deteriorating despite a greater or lesser improvement in the functional status of patients after a stroke^6^. Therefore, this condition should be systematically monitored and included in the assessment of all results of clinical trials on strokes, and activities related to primary or secondary prevention of dementia should be implemented^48^. Previous studies show the effectiveness of introduced cognitive and functional training^5,7,49,50^. Interventions aimed at primary and secondary prevention of dementia can reduce the risk of developing demantia and thus increase the chance of improving the functional status and regaining independence in the process of recovery and rehabilitation of stroke patients.

To sum up: Knowledge of the cognitive state of patients after a stroke may be the key to improve their functional status, better rehabilitation outcome and regaining independence in basic everyday activities. Setting realistic goals by the medical staff and using modified therapeutic procedures adapted to the patient's cognitive abilities after a stroke may translate into a rehabilitation and functional outcome. Therefore, in the holistic model of rehabilitation of patients after a stroke, the cognitive state should also be considered in the entire rehabilitation and treatment process.

Limitations. The authors are aware of some limitations of the presented studies. First, the division into groups was based on a screening test, not a diagnosis. We do not know the cognitive state of the pre-stroke period. Although one of the exclusion criteria from the study was the presence of pre-stroke dementia, it cannot be ruled out that such symptoms had already occurred before but were not diagnosed. It is a single-centre study, so the results must be interpreted cautiously for other populations.

## Conclusions

The lowest improvement in functional status was noted in post-stroke patients with moderate dementia.

Cognitive impairments occurring at the time of admission to the ward and at discharge, as well as the functional status at the time of admission to the ward, may be factors determining the patient's functional status at the time of discharge from the ward. Still, they are not factors preventing the improvement of the functional status of patients after a stroke.

## Data Availability

Te datasets used and/or analysed during the current study available from the corresponding author on reasonable request.
